# Intergenerational Solidarity in Appalachian Ohio: Exploring Social Connectedness and Extreme Weather Response

**DOI:** 10.1093/geroni/igaf122.1244

**Published:** 2025-12-31

**Authors:** Fiona Doherty, Smitha Rao, Holly Dabelko-Schoeny

**Affiliations:** University of Tennessee, Nashville, Tennessee, United States; Ohio University, Columbus, Ohio, United States; The Ohio State University, Columbus, Ohio, United States

## Abstract

Older adults (aged 65+) and emerging adults (aged 18-29) face vulnerability to two converging crises: social disconnectedness and extreme weather attributable to climate change. These challenges compound each other, creating a cycle of risk. Disconnected communities struggle to respond collectively to extreme weather, increasing the likelihood of harm. Additionally, extreme weather can disrupt social connectivity by altering local environments. While older and emerging adults share vulnerabilities and agency, generational divides hinder cooperation, highlighting the need to build intergenerational capacities and connections. This study employed an intergenerational solidarity framework in Athens County, a rural and underserved area in Appalachian Ohio, to 1) compare older and emerging adults’ experiences of social connectedness and extreme weather, and 2) examine outcomes from intergenerational and intragenerational conversations focused on enhancing social connectedness and extreme weather adaptive capacity. Sixteen older and emerging adult participants engaged in Participatory Photo Mapping from May to September 2024. Data collection included generating photos and spatial maps of social connectedness factors and conducting photo-and-map elicitation interviews and focus groups. Data analysis included iterative thematic and comparative analyses. Older adults’ connectedness assets were closer to home while emerging adults’ assets were dispersed across the county. Older adults were more experienced and confident in handling extreme weather situations. In the intergenerational focus group, participants valued the other generation’s perspectives. Conversely, the intragenerational group showed a mix of curiosity and skepticism about the other generation’s contributions to extreme weather response. Findings highlight significant potential for knowledge exchange and trust-building through participatory intergenerational dialogues.

